# Language, identity, and survival: an ethnographic study on the revitalization of the Limola language in South Sulawesi

**DOI:** 10.3389/fsoc.2025.1686828

**Published:** 2025-11-13

**Authors:** M. Nur Hakim, Wahyu Hidayat, Jusrianto Jusrianto

**Affiliations:** Faculty of Teacher Training and Education, Universitas Cokroaminoto Palopo, Palopo, South Sulawesi, Indonesia

**Keywords:** Limola language, language revitalization, ethnolinguistics, intergenerational transmission, digital heritage

## Abstract

The Limola language, spoken in Sassa Village, Luwu Utara Regency, South Sulawesi, represents an essential cultural identity and intangible heritage element. However, its transmission has declined due to generational language shift, placing it at risk of endangerment. This study explores the roles of community actors and strategies in revitalizing the Limola language across traditional, domestic, and digital domains. Using a qualitative ethnographic approach, data were collected through in-depth interviews with native speakers, participatory observation during traditional rituals, and documentation of digital content initiatives. Thematic analysis was applied to identify patterns of language use and preservation practices. The findings reveal that the Limola language plays a central role in various traditional rituals, such as funeral ceremonies, harvest festivals, and *Mopacci*, while still used to a limited extent in everyday communication. Traditional leaders maintain Limola through ritual use, native speakers—particularly elders—serve as intergenerational transmitters in family contexts, and youth utilize social media to promote the language creatively. This multi-domain revitalization approach integrates cultural traditions with modern technological adaptation, contributing to language vitality while strengthening community identity. The results highlight the theoretical significance of language as symbolic capital, the practical value of community-led digital engagement, and the policy need for formal support in education and cultural heritage recognition. This study recommends sustaining language revitalization through integrated, participatory strategies that combine ritual, domestic, and digital practices.

## Introduction

Language is a fundamental pillar in forming social identity, cultural expression, and the transmission of knowledge across generations. However, in this era of globalization and the rapid expansion of information technology, the vitality of regional languages faces unprecedented pressure. According to UNESCO (2021), (2023), nearly 40% of the world's approximately 7,000 languages are endangered, with one language disappearing every 2 weeks. This decline is not a natural process but rather the outcome of complex historical, social, political, and economic forces. As Pine and Turin (2017) note, language loss is frequently linked to colonization, the marginalization of Indigenous communities, and state language policies that prioritize national or dominant languages over minority ones. Consequently, the extinction of languages reflects not only a communicative crisis but also a profound erosion of cultural identity and sovereignty. In response, revitalization efforts must be conceptualized as holistic, addressing structural inequalities and empowering communities to reclaim their linguistic heritage.

In the context of revitalization, it is evident that language preservation efforts are aimed at producing more speakers and creating space for cultural revival, collective healing, and strengthening social bonds within communities (Pine and Turin, 2017). Therefore, language revitalization must be viewed as a holistic process involving education, politics, and active and conscious community participation. In line with this, language as a tool for cultural transmission must also be supported by systematic documentation and socialization efforts so that its cultural values can continue to be passed on to future generations (Khalsiah et al., 2025). As Davis (2015) emphasized, language revitalization is not only about restoring language skills but also strengthening ethnolinguistic identity and expanding community participation through language affiliations involving speakers, learners, and non-speaker supporters.

In Indonesia, as a country with extraordinary linguistic diversity, 718 regional languages have been identified, making preserving regional languages a crucial challenge. Data from the Language Development and Guidance Agency (2024) shows that 11 languages are extinct, five are critically endangered, and 25 are endangered. Factors such as the dominance of the national language, urbanization, marginalization of indigenous communities, and changes in communication patterns influenced by social media are accelerating the erosion of local languages (Grenoble and Whaley, 2006; Crystal, 2000). More than 300 ethnic groups and 700 local languages contribute unique traditions and philosophies, enriching Indonesia's national identity. This implies that preserving local languages is an inherent part of efforts to revitalize culture and national identity (Redjeki et al., 2025). One important indicator of a language's vitality is its presence in public spaces. The representation of local languages in street signs, road signs, vocabulary records, folk tales, and other visual symbols is a form of symbolic recognition that can foster collective pride in preserving linguistic heritage (Benu et al., 2022; Gorter, 2006; Darquennes, 2007).

However, the challenges of revitalization are not only technical or structural. As mentioned by Albury (2015), handing over full responsibility for revitalization to indigenous communities often becomes a burden, especially if there are differences in perceptions about the form of linguistic empowerment. Additionally, the dominance of majority-language media further drives language shift (Eisenlohr, 2004). In line with this, Hatzikidi et al. (2021) emphasize that state power often poses a serious threat to cultural identity, while minority communities are often insufficiently strong to protect their languages without structural support.

Despite the growing body of research on language revitalization, few studies in Indonesia have ethnographically examined how traditional, domestic, and digital domains intersect to sustain endangered languages at the community level. Moreover, the roles of different social actors—elders, parents, and youth—in shaping revitalization practices remain underexplored. This study addresses these gaps by focusing on the case of Limola, an endangered language in South Sulawesi.

One case that reflects this situation is Limola, a regional language traditionally used by indigenous communities in Sassa Village, North Luwu Regency, South Sulawesi. Based on the results of participatory observation and in-depth interviews with some key informants, including traditional leaders, youth, and native speakers, it is known that Limola is endangered. Its use in everyday life is increasingly limited and generally only survives in the context of traditional ceremonies or certain rituals. Meanwhile, the younger generation prefers to use Indonesian, Toraja, or colloquial language, which is heavily influenced by social media, indicating a rapid and significant language shift. However, it is important to understand that using the first language is crucial as a bridge to understand new concepts in the second language (Wuttiphan et al., 2025) and as a means of intergenerational transmission (Hermes et al., 2012). The loss of the first language can significantly impact the younger generation's cognitive processes and identity development.

This situation not only indicates a decline in the number of Limola speakers but also weakens the social function of the language. Limola plays a central role in the local cultural structure, particularly in harvest ceremonies, traditional weddings, and traditional rituals. Various forms of cultural expression, such as mantras, songs, and traditional prayers, can only be authentically conveyed in Limola. As indicated by Yulianti et al. (2024), Limola has a rich and flexible syntactic structure, reflecting the diversity of linguistic functions closely linked to the cultural meanings of the Sassa community. Although the number of speakers remains relatively high in some villages, the decline in usage among younger generations has placed the language in an endangered status. This indicates the importance of comprehensive revitalization efforts, including syntactic documentation and integration into formal education to ensure sustainable transmission. Therefore, the loss of this language will directly impact the extinction of important elements in the knowledge system and cultural practices of the local community (Hornberger and King, 1996). In line with this, Michael et al. (1998) show that indigenous communities that have successfully preserved their language and cultural practices have higher levels of social and psychological resilience, confirming that language is a means of communication and the foundation of cultural survival. Furthermore, White (2002) and Hinton et al. (2018) emphasize that the use of mother tongue as a symbol of authenticity and collective identity is strengthened through language use at home (language nesting). Thus, efforts to revitalize the Limola language are aimed at preserving a means of communication and strengthening a sense of belonging and cultural authenticity amid the pressures of global homogenization.

In addition, efforts to revitalize regional languages also require intervention through formal education, particularly at the early childhood education level. DePalma et al. (2018) emphasized that teachers play a strategic role as agents of language revitalization, primarily through community-based learning activities and in-depth sociolinguistic reflection. Teachers not only act as instructors who transmit linguistic skills, but also as role models in fostering positive attitudes toward regional languages as symbols of cultural identity continuity. Teacher education programs designed in a participatory manner can enhance awareness of sociolinguistic realities in society and strengthen teachers' ability to support using minority languages in daily interactions at school. Gorter (2012) emphasized that researchers and academics also have a social responsibility to support policies and efforts to revitalize minority languages, ensuring they remain relevant and valuable to society. This approach demonstrates that the success of language revitalization, including Limola, is not solely determined by preserving traditional rituals but also by transforming the formal education domain, which fosters language pride from an early age.

This study is closely related to various previous studies on language revitalization. Grenoble and Whaley (2006) emphasize that revitalization is aimed at maintaining the number of speakers and restoring the social function of language in various domains, both cultural and formal. This is relevant to the condition of the Limola language, which is currently mostly used in traditional rituals, while in everyday life it has been replaced by other languages. In addition, recent research in Indonesia has also shown various creative and complementary approaches to revitalization. Mulyawan (2021) shows that language revitalization in public spaces, such as using Balinese script on signboards and signage, can be an effective symbolic strategy to strengthen cultural identity and increase the visibility of local languages. Therefore, efforts to revitalize the Limola language must combine various strategies: strengthening traditional communities, integrating the language into the formal education system, fostering cultural creativity, and optimizing public spaces as domains for language use and pride. This integrated approach is expected to comprehensively address the revitalization challenges while affirming the Limola language as a living and sustainable collective identity.

Considering the strategic role of the Limola language as a medium of cultural expression and collective identity, this study aims to examine efforts to revitalize the Limola language and the social context of its use, as well as to analyze the role of traditional leaders, native speakers, and the younger generation through an ethnographic approach. This study traces the socio-cultural dynamics that influence the survival of the Limola language and identifies community-based revitalization strategies that have been or can be developed to ensure the sustainability of the language while preserving the ethnolinguistic identity of the Sassa community. The findings of this study are expected to contribute conceptually and practically to the discourse on regional language preservation in Indonesia, while enriching models of language revitalization based on community participation.

This study contributes to the field by offering an ethnographically grounded analysis of community-led language revitalization that integrates ritual, domestic, and digital practices. It also develops a contextualized understanding of how different social actors collaborate in sustaining language use and identity, thereby enriching theoretical and practical approaches to endangered language revitalization in Indonesia.

Based on the background and objectives presented above, this study is guided by the following research questions:

1. How is the Limola language revitalized across different domains—traditional rituals, domestic settings, community activities, and digital media—within the Sassa Village community?
2. What roles do traditional leaders, native speakers, and the younger generation play in preserving, transmitting, and adapting the Limola language in a changing sociocultural context?
3. How do socio-cultural dynamics shape the sustainability and intergenerational transmission of the Limola language as an element of cultural identity?

## Literature review

Language revitalization has been widely discussed in global and Indonesian contexts, highlighting diverse strategies and outcomes. Globally, outline revitalization as a multifaceted process aimed not only at increasing the number of speakers but also at restoring a language's social functions across different domains (Farfan and Cru, 2021). The role of community-driven initiatives, intergenerational language transmission, and institutional support in reversing language shift (Nurman et al., 2022). Case studies such as the revitalization of Māori in New Zealand and Welsh in the United Kingdom demonstrate the importance of integrating language education into formal schooling, promoting domestic language use, and leveraging media to enhance visibility and prestige.

In Indonesia, language revitalization efforts have focused on symbolic visibility, policy integration, and community participation. The use of Balinese script in public spaces reinforces cultural identity and fosters pride among speakers (Purnawati et al., 2025). The preservation of endangered languages through the documentation of oral traditions (Yusriadi et al., 2022), the linguistic landscape as a key driver of language vitality in urban settings Benu et al. (2022). Despite these efforts, many regional languages continue to face severe endangerment due to intergenerational transmission breakdown, urban migration, and the dominance of Indonesian in formal domains (Nurman et al., 2022). Furthermore, this approach is also based on the theory of language as a social practice developed by Bourdieu (1991). Within this framework, language is viewed as a means of communication and symbolic capital that reflects and reproduces social structures, power, and identity.

While existing research provides valuable insights, few studies have ethnographically explored how community actors across generations work together to revitalize a language within a localized context. Moreover, there remains limited research on how traditional, domestic, and digital practices intersect to sustain language vitality. This study addresses these gaps by examining the Limola language in Sassa Village, focusing on the roles of traditional leaders, native speakers, and youth in maintaining language use and identity in a changing sociocultural landscape.

### Theoretical framework

This study is grounded on (Bourdieu, 1991) concept of language as symbolic capital, which views language not merely as a communicative tool but as a form of social power that reflects and reproduces social structures, cultural identity, and power relations. Within this framework, revitalizing the Limola language involves more than preserving vocabulary and grammar; it entails reclaiming symbolic power and re-establishing language as a marker of collective identity.

Additionally, this study draws ecological approach to language revitalization, which posits that language vitality depends on interconnected domains, including home, school, community, and media (McCarty, 2018). By examining how the Limola language functions within traditional rituals, domestic interactions, and digital platforms, this study explores how these domains interact to sustain language use. The framework also aligns with Farfan and Cru (2021) notion of language affiliation, highlighting how language revitalization efforts build community participation and identity beyond the speaker population.

Together, these theoretical perspectives guide the analysis of how the Limola language is preserved, transmitted, and transformed across generations, while revealing how power, identity, and cultural continuity shape language revitalization in a rapidly changing world.

## Methods

### Research design

This study uses a qualitative approach with ethnography as the main framework to explore social, linguistic, and cultural practices related to transmitting the Limola language in the context of the Sassa community in North Luwu. Field research was conducted from June to July 2025, comprising approximately 1 month of ethnography in the field and 1 month of follow-up online engagement. The research team consisted of three researchers with backgrounds in education, including one bilingual facilitator familiar with local customs and Limola terminology. This approach was chosen because it aims to understand social reality from an emic perspective, that is, from the point of view of insiders (Limola speakers themselves), which cannot be achieved through quantitative or etic approaches that are separate from the local cultural context.

This ethnographic design is aligned with the study's theoretical grounding detailed in the Literature Review and Theoretical Framework section. Therefore, transmitting the Limola language is not merely about preserving vocabulary and grammatical structures, but also involves negotiations of cultural identity, intergenerational power relations, and responses to the pressures of modernization.

This study is based on the concept that language is a cultural tradition that shapes human perceptual, cognitive, and interpretive capacities (Joseph, 2004). Within this framework, language functions as a means of conveying meaning and plays a vital role in affirming social membership, reinforcing shared values, and fostering collective pride. Amidst the tide of globalization and modernization, the Limola language has become a symbol of resistance against cultural homogenization while also serving to strengthen local communities' identity.

Thus, the ethnographic approach allows researchers to delve into symbolic and affective dimensions of linguistic practices. Whether they occur in everyday conversations, traditional ceremonies, or oral narratives passed down from generation to generation. This study also seeks to document how the Sassa community constructs meaning from their language and how the Limola language is a battleground between collective memory, modernity, and social change. We acknowledge our positionality as collaborator researchers with prior experience in community work in South Sulawesi. This position facilitated access and trust with community members, while also requiring continuous reflexivity to mitigate interpretive bias. To address this, we maintained a reflexive journal, conducted iterative sense-making discussions with community representatives, and used peer debriefing within the team to challenge assumptions and check interpretive coherence.

Before fieldwork, the research team conducted an initial literature review to identify theoretical frameworks, prior studies, and methodological considerations relevant to minority language revitalization. Research permits were obtained from the local government and traditional leaders, and an explanation of research objectives and methods was provided to community representatives. Ethical clearance was ensured by preparing informed consent forms in Indonesian and Limola (oral explanation), guaranteeing anonymity and allowing participants to withdraw at any stage without consequence. Ethical approval was obtained from the Cokroaminoto Palopo University Research Institute. Verbal consent was obtained in Indonesian, with guarantees of anonymity, the right to withdraw at any time, and permission for photographs. The research plan included a detailed timeline covering preparation, participant recruitment, data collection, transcription, coding, thematic analysis, triangulation, and report writing.

### Research site and participants

The research location is Sassa Village, Baebunta Subdistrict, North Luwu Regency, South Sulawesi Province, Indonesia. This village is known as one of the centers of the Limola language community. Informants in this study consisted of traditional leaders, native speakers, and young people involved in cultural or educational activities. Selection criteria include proficiency in the Limola language, involvement in cultural preservation activities, and experience in education or community organization. More specifically, participants were required to (1) demonstrate active use or deep understanding of the Limola language in daily communication or ritual contexts; (2) have experience participating in or leading traditional ceremonies, language-related educational programs, or digital revitalization initiatives; and (3) represent different generational and social roles within the community. All participants provided informed consent prior to participation.

Participant selection was conducted using purposive sampling to ensure the inclusion of individuals with deep knowledge of the Limola language and culture. The recruitment process began with mapping the community's social structure, identifying key figures, and consulting local gatekeepers to gain trust. Participants were grouped into three main categories: (1) Traditional leaders–active in leading cultural ceremonies and custodians of oral traditions; (2) Native speakers–primarily middle-aged and elderly individuals who use Limola regularly in domestic or community settings; and (3) Younger generation–youth involved in cultural groups or educational programs, including those using digital media for language promotion. A total of 24 participants were involved in this study, consisting of 6 traditional leaders (aged 60–75), 10 native speakers (aged 40–65), and 8 youth participants (aged 18–30). Gender distribution included 14 males and 10 females, ensuring balanced representation of perspectives. The diversity of participants across age groups, social roles, and linguistic competencies allowed the study to capture a more comprehensive picture of language revitalization practices in the community. The number of participants was determined based on data saturation, with interviews and observations continuing until no new significant information emerged.

In total, we conducted 24 interview sessions, corresponding to one in-depth interview with each participant. Each interview lasted 45–90 min and was conducted in locations familiar and comfortable to the participants. In addition, 3 follow-up interviews were conducted with key informants (traditional leaders and youth coordinators) to clarify emerging themes and validate interpretations. Participatory observation was carried out over approximately 120 h, encompassing 8 major events (including mopacci, motuno walundaka, and harvest rituals) and 15 household visits. These extended engagements enabled the researchers to observe language use in both ceremonial and everyday contexts, providing rich, contextualized insights into the dynamics of Limola language revitalization.

### Data collection techniques

Data was collected through two main techniques: (1) in-depth interviews and (2) participatory observation. Interviews were conducted in a semi-structured manner to allow flexibility in exploring personal narratives, perceptions of the sustainability of the Limola language, and intergenerational experiences related to language inheritance. The interview guide covered topics such as language use at home, in traditional rituals, and perceptions of the cultural value of the Limola language. Participatory observation was conducted in various community activities, including traditional ceremonies and daily interactions at home. The procedures were performed as follows:

(1) Preparatory Phase: The research team developed an interview guide in Indonesian, with potential key terms in Limola, to encourage respondents to answer in their preferred language. To refine question clarity, a pilot test of the guide was conducted with two non-sample participants.
(2) Participatory Observation: Researchers attended major cultural events such as *mopacci* (a pre-wedding purification ritual), *motuno walundaka* (a mourning rice-burning ritual), and harvest festivals. During these events, detailed field notes were taken to capture linguistic choices, gestures, participants' roles, and contextual cues. Audio recordings were made when permitted, ensuring minimal disruption to natural interactions.
(3) In-depth Interviews: Each session lasted between 45 and 90 min and was conducted at locations comfortable for participants. The interviews began with rapport-building and moved from general questions to more specific ones about language practices, challenges, and revitalization strategies.
(4) Community Documentation: The study analyzed publicly available content from the *Rumpun To Limola* community's Instagram account (@mosambuakeofficial), cataloging vocabulary, idiomatic expressions, and cultural concepts portrayed in posts, videos, and captions.
(5) Digital Ethnography: Instagram Sampling and Coding: We analyzed posts from the community account @mosambuakeofficial during the period June–July 2025. Inclusion criteria included: (a) explicit use of Limola in captions/audio/text overlays; (b) relevance to cultural practices or language promotion; and (c) public accessibility. Exclusion criteria included duplicate posts or posts without linguistic content. A corpus of posts was compiled.

Posts were imported into NVivo 15 and coded inductively for (1) language functions (e.g., vocabulary teaching, storytelling, ritual narration), (2) cultural values, and (3) engagement cues (e.g., invitations to practice, intergenerational appeals). Two coders independently tested the shared code book, resolving disagreements through discussion. The refined code book was then applied to the complete dataset.

### Data analysis

The data were analyzed using a thematic analysis approach developed by Braun and Clarke (2006), which emphasizes the active process of researchers in identifying, analyzing, and reporting patterns (themes) in the data. This analysis was conducted through six stages: (1) familiarization with the data through transcription and repeated reading, while noting initial ideas; (2) systematically generating initial codes across the entire data set; (3) identifying themes by grouping codes into potential themes; (4) reviewing themes to ensure internal coherence and relevance to the overall data; (5) clearly defining and naming themes; and (6) producing a final report that presents a thematic narrative supported by data quotations.

All interviews were transcribed verbatim, including non-verbal expressions where relevant. Field notes from observations were digitized and merged with transcripts in NVivo software to facilitate coding. Codes were applied inductively, allowing patterns to emerge organically from the data rather than imposing pre-defined categories. Codes were then grouped into broader themes through iterative comparison. The themes were refined through peer debriefing sessions within the research team to check for consistency and eliminate overlaps.

To enhance the credibility of the analysis, source triangulation (interviews, observations, and/or local dictionaries) was conducted, and key informants were checked to verify interpretations. For example, interpretations of the *mopacci* ritual vocabulary (interview with participant TL-03, a traditional leader aged 72) were cross-checked with fieldnotes from a *mopacci* ceremony observed on 12 June 2025 and a related Instagram post published on 20 June 2025 by the @mosambuakeofficial community page. The post featured a video clip of the *mopacciritual* accompanied by the repeated use of the term “*mappangolo*,” which refers to the symbolic act of cleansing and spiritual preparation before marriage. Convergence across these sources strengthened the interpretation of *mappangoloas* a culturally salient key word that embodies not only purification but also the transmission of ancestral blessings and collective values tied to kinship and marital harmony. To enhance the credibility of the analysis, source triangulation (interviews, observations, and/or local dictionaries) was conducted, and key informants were checked to verify interpretations. Data processing was also supported by NVivo software, which assisted in the systematic coding and grouping of data in line with the principle of recursive process.

The final stage involved synthesizing the findings into a coherent narrative, integrating direct quotations from informants with analytical commentary, and linking them to relevant theoretical and empirical literature. The report was structured to reflect the three major themes identified—revitalization efforts, social context of use, and roles of community actors—followed by a discussion of implications for policy and practice. Recommendations were formulated in consultation with community stakeholders to ensure feasibility and cultural appropriateness.

### Findings

#### Theme 1: efforts to revitalize the limola language

This study found that the Limola language is still actively used in various cultural contexts by the Sassa Village, North Luwu Regency community. This language is not merely a means of communication, but also a medium for expressing spiritual, social, and cultural values passed down from generation to generation. Various forms of revitalization have emerged in various areas of community life, as summarized in the following Table 1.

**Table 1:** Dominant use of Limola language in the Limola community.

**Dominant**	**Language function**	**Main actors**	**Form of revitalization**
Traditional ritual	Delivery of prayers, advice, and symbolic meanings	Traditional leaders, elders	*Mopacci, Motuno Walundaka* ceremonies
Domestic environment	Daily communication, child rearing	Housewives, families	Informal language transmission at home
Digital media	Vocabulary education, cultural transmission, and entertainment	Youth community	Instagram content, educational videos
Community interaction	Strengthening local identity, collective cultural expression	All residents	Culturally-based community activities

One of the most prominent forms of revitalization can be seen in using Limola language in traditional rituals. This language is used in important ceremonies such as *mopacci*, part of the traditional wedding procession, where language becomes a vehicle for conveying prayers, life advice, and the symbolic values of each procession stage. This practice underscores the role of the Limola language as a medium for spiritual meaning and collective morality.

The same thing happens in mourning rituals, such as *motuno walundaka* (the ritual of burning rice in bamboo), where the language is used for this activity. In this context, Limola connects life and death and becomes a vehicle for passing on values across generations. These two rituals show that language lives in every community life phase: birth, marriage, and death.

Beyond the ritual realm, the domestic environment is crucial in preserving the Limola language. Women, particularly housewives, actively use this language in child-rearing and daily communication. The language is organically transmitted through these informal practices within the private family sphere. This underscores that revitalization does not solely rely on formal institutions but also depends on the role of the community, especially women, in maintaining linguistic continuity.

Revitalization strategies are adapted in the digital space, as done by the *Rumpun to Limola* community through their Instagram account @mosambuakeofficial. They produce creative content in Limola through short videos, quotes, and cultural dialogues that introduce vocabulary and grammatical structures, while instilling local cultural values. This initiative reflects efforts to reach the younger generation through media they are familiar with, as well as to expand the domain of language use to the public and virtual spheres.

Thus, the revitalization of the Limola language does not take place in a single channel, but through various complementary spheres of life: sacred, domestic, community, and digital. Each sphere contributes to language preservation, both symbolically and practically. As Pine and Turin (2017) emphasized, effective language revitalization requires a holistic approach that combines cultural strength, community participation, and technological adaptation. Limola language, in this context, has demonstrated its vitality through the continued social and cultural functions it serves in the lives of the Limola community.

This finding supports Bourdieu (1991) conception of language as symbolic capital, where linguistic practices in ritual, domestic, and digital spheres not only serve communicative purposes but also reproduce social hierarchies, cultural authority, and collective identity. The persistence of Limola across these complementary domains shows that revitalization is not a linear process but an ongoing negotiation between tradition and modernity, deeply embedded in power relations and community agency. This highlights that revitalization success depends not merely on linguistic transmission but also on maintaining the symbolic value of the language in diverse social contexts.

#### Theme 2: Social context of limola language use

Limola is predominantly used in various traditional activities that are an important part of the cultural life of the Sassa community. For example, in harvest festivals, traditional wedding ceremonies, mourning ceremonies, and other traditional rituals. Limola language is the primary medium for conveying prayers, chants, traditional songs, and ancestral wisdom. This demonstrates that the Limola language holds strong symbolic and spiritual significance within the community's cultural context. Beyond its ceremonial functions, the Limola language is occasionally still used in everyday communication, particularly when both speakers have the same language proficiency and ethnolinguistic backgrounds. However, its use in informal settings is limited and highly situational, depending on the social context and the relationship between speakers. This condition indicates that the Limola language is still alive in the community's collective memory, even though it is not the primary means of communication in everyday life.

To further understand the use, meaning, and vitality of the Limola language in the context of daily life and customs, this study uses data obtained through ethnographic observation, semi-structured interviews, and questionnaires with community members, including traditional elders, native speakers, and several youth leaders. The following section presents selected quotations and observational data illustrating the social and linguistic functions of the Limola language in various situations, both formal and informal, while also showing how this language is maintained, negotiated, and transmitted across generations.

“*Based on participatory observation, the Limola language is currently predominantly used in traditional ceremonies, such as mourning ceremonies, where one of the activities is motuno walundaka [burning rice using bamboo]. This traditional activity is carried out on the fourth night of the mourning ceremony to be presented to the traditional leaders. The activity includes women and men, each performing their respective tasks. Specifically, the women are responsible for filling the bamboo with rice, while the men and teenage boys handle the burning. During the motuno walundaka activity, the speakers use the Limola language. Some of the terms used in the activity include mosuda [sit], mande [eat], moinu [drink], mojama [majama], malossu [hot], totora [stand], and various other terms used in the traditional ceremony.”*

Furthermore, based on observations of wedding activities, the people of Sassa Village still practice the *mopacci* tradition before the wedding ceremony. This tradition is similar to those found in other areas of South Sulawesi, such as the Bugis Makassar culture, which is known as *Mappaci'*, a form of spiritual and physical purification for the bride and groom. The *mopacci* ceremony is attended by various figures, such as traditional leaders, village heads, hamlet heads, and multiple members of the local community in Sassa Village. Different terms in the Limola language are used in the mopacci ritual, such as: *Alonga* [pillow], *Seseri sabbe* [silk sarong], *Colli tawe punti* [banana leaf], *Tawe panasa* [jackfruit leaf], *Tawe pacci* [pacar leaf], *Wia kunnyi* [yellow rice], *O‘damba* [Candle], *Pambolia/pesaa* [Container/place], *Uwai* [Water]. The use of the Limola language in traditional activities is still strongly preserved by the community in Sassa Village.

Limola language is not only limited to traditional mourning activities and *mopacci'* ceremonies, but harvest festivals also continue to preserve Limola language in communication. This was revealed in interviews with KS informants, who stated that:

“*Yang diadakan pesta panen, itu pesta panen padi. Ini sebagai bentuk Syukur atas hasil panen. Ada kegiatan adat itu moroda, ma balole, banyak kegiatan lain, ada juga permainan gasing ada tim sando dan tim pengaroh. Kalau kegiatan adatnya itu pakai bahasa Limola, tapi kalau permainnya sudah bercampur bahasa, karena banyak yang terlibat”*.

[The harvest festival held is a rice harvest festival as a form of gratitude for the harvest obtained. During this event, there are various traditional rituals such as *moroda* and *ma balole and* other activities, including traditional games such as spinning tops, with teams of sando and pengaroh. For traditional rituals, the community uses the Limola language exclusively, while for games and other activities, the language used is mixed, as many participants come from different backgrounds].

In addition to serving as a ceremonial language, Limola is sometimes still used in everyday communication, especially when both speakers have the same language skills and ethnolinguistic backgrounds. This was conveyed by informant SP (age 46), who explained:

“*Orangna sajitia [saudara], balibolaku [tetangga], poko' hari-hari kalau ketemu pake bahasa limola, itu kakak tadi pakai bahasa limola. Kalau sama-samaki orang Limola pasti kalau ketemu pakai bahasa Limola, kecuali kalau ada orang pendatang tidak mengerti bahasa Limola baru pakai bahasa Indonesia. Biasanya juga pendatang kalau menikah dengan orang sini lama-lama juga bisa berbahasa Limola”*.

[People such as family, relatives, and neighbors usually speak Limola when they meet daily. If they are all Limola speakers, they will speak Limola when they meet, unless there are outsiders who do not understand, in which case they will use Indonesian. Usually, migrants who marry local people will also learn to speak Limola over time].

These findings indicate that the survival of the Limola language depends not only on preservation in traditional spaces but also on everyday practices that occur naturally among communities with similar ethnolinguistic backgrounds. The acknowledgment of informants such as SP confirms that this language is still used in casual conversations in homogeneous social environments, especially among native speakers. Moreover, linguistic acculturation occurs when migrants marry residents and gradually master Limola. This phenomenon demonstrates that a language remains alive if there are spaces for interaction that encourage its use. Therefore, revitalization efforts must continue to focus on symbolic preservation and creating social conditions that enable the language to be used functionally, familiarly, and sustainably in daily life.

Viewed through the lens of language ecology (Eliasson, 2015), these findings illustrate that the vitality of Limola depends on the interplay between environmental factors (social settings), speaker attitudes, and intergroup relations. The situational use of Limola—whether in rituals, households, or community interactions—demonstrates how language choices are shaped by social structures and power dynamics. The adaptation of outsiders who learn Limola after intermarriage also reflects a form of bottom-up language expansion, showing that linguistic revitalization can emerge organically when social networks are inclusive and sustained.

#### Theme 3: The role of traditional leaders, native speakers, and the younger generation

The revitalization of the Limola language cannot be separated from the active role of various elements of society involved in preserving and transmitting this language in everyday life and a cultural context. Field findings show that traditional leaders, native speakers, and the younger generation each contribute to preserving and inheriting the Limola language.

**Figure 1:**
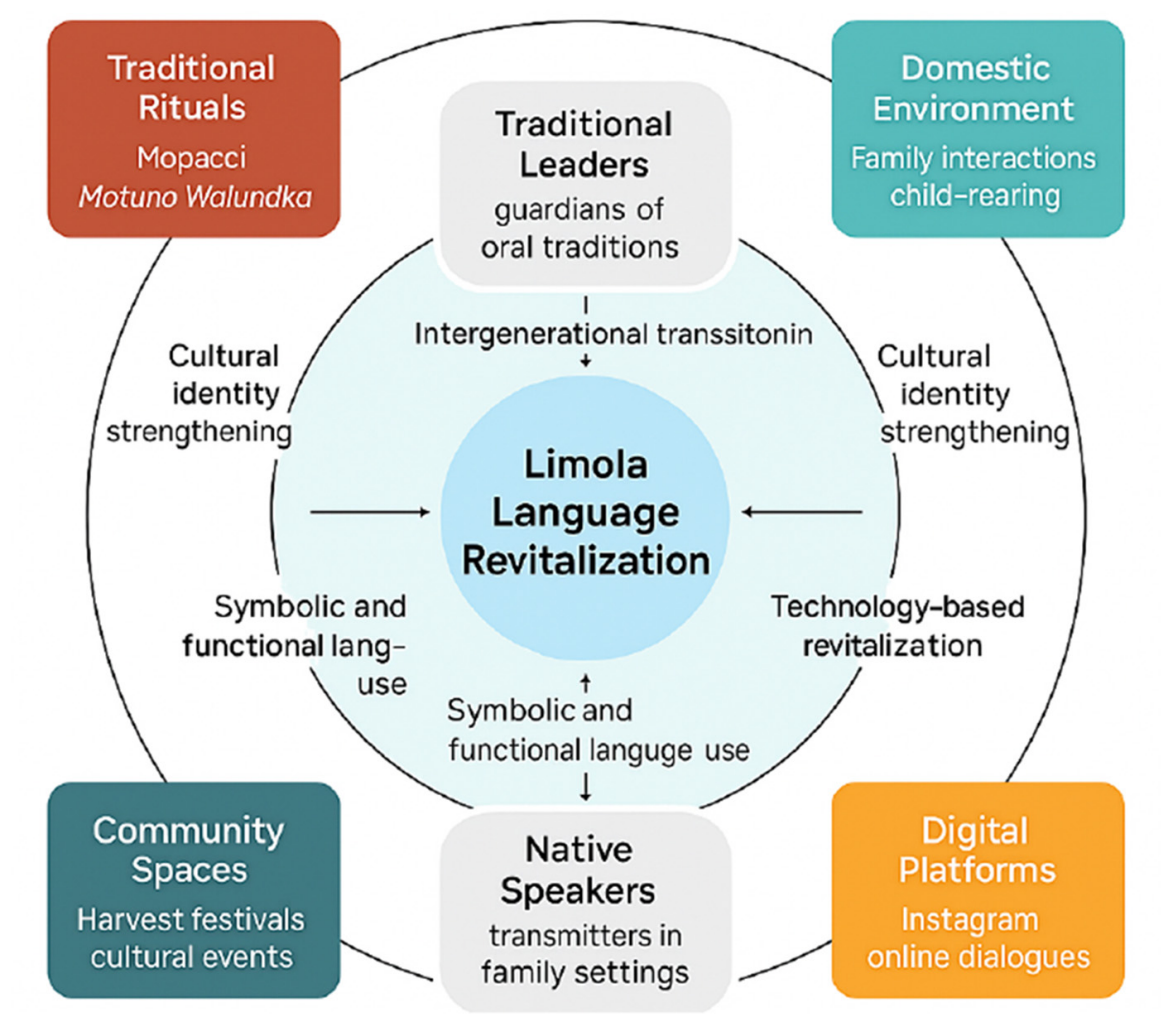
Conceptual model of Limola language revitalization showing the interaction between key domains, community actors, and revitalization strategies in Sassa village.

From a sociolinguistic perspective, the active engagement of youth aligns with Eliasson (2015) model of reversing language shift, which emphasizes the need to re-establish intergenerational transmission in both traditional and modern domains. However, the findings also reveal contradictions and structural limitations: while digital initiatives broaden visibility and participation, they risk commodifying cultural practices if not grounded in deeper cultural knowledge. Moreover, the absence of institutional support, such as formal learning spaces, remains a critical barrier. These tensions suggest that revitalization requires multi-level interventions—combining grassroots creativity with policy-level support—to ensure authenticity, sustainability, and scalability (see Figure 1).

### The role of traditional leaders as guardians of oral traditions and culture

Traditional leaders play a central role in preserving the Limola language, particularly through their involvement in various traditional ceremonies such as harvest festivals (*mappadendang*), mourning ceremonies, traditional weddings (*mopacci*), and other local rituals. In these activities, the Limola language is the primary language for conveying prayers, advice, incantations, and traditional narratives with symbolic and spiritual meanings. This role positions traditional leaders as the authoritative guardians of oral traditions passed down through generations.

An interview was conducted with one of the traditional leaders, Mr. SD, aged 71. Mr. SD also previously served as the Chairman of the Traditional Council in Sassa Village.

“*Misalnya ada kegiatan adat, itu pakai bahasa Limola, ada yang mau dijatuhi sanksi atau hukum adat, itu pakai bahasa Limola. Misalnya kalau di Bugis itu, ada Tudang Sipulung atau musyawarah ada mau diputuskan itumi di sini bahasa Limola dipakai. Kalau pesta panen juga melibatkan dewan adat, itumi bahasa Limola juga dipakai”*.

[If there are traditional activities, Limola is always used. This also applies when imposing sanctions or enforcing customary law. For example, the *Tudang Sipulung* tradition in Bugis society, a traditional deliberation to make decisions, is also conducted using Limola. During harvest festivals involving the traditional council, Limola is still used].

An interview with Mr. SD, a 71-year-old traditional leader and former head of the Sassa Village Council, revealed that the Limola language plays a central role in every aspect of traditional life. The use of Limola in various traditional activities, ranging from funeral ceremonies, traditional weddings (*mopacci*), harvest festivals (*mappadendang*), to traditional deliberations, shows that this language is not merely a means of communication, but also a vehicle for conveying the spiritual values, ethics, and social norms of the Limola community. Additionally, the involvement of traditional leaders in customary legal activities, such as imposing sanctions, highlights their authority in preserving the authenticity of traditional practices. By using the Limola language as the primary medium, traditional messages can be conveyed in their entirety without losing their symbolic meaning. Furthermore, Mr. SD revealed that:

“*Anak-anak sekarang banyak juga yang tidak paham, jadi kami harus terus pakai supaya mereka dengar dan terbiasa. Saya juga sering ajak pemuda-pemuda terlibat kegiatan adat, seperti pesta panen, acara motuno walundaka atau membakar nasi menggunakan bambu, mereka bisa ikut. Dengan ikut acara-acara, mereka perlahan-lahan bisa mengerti bahasa Limola dan nanti bisa diteruskan. Kalau hilang ini bahasa, bukan cuma kata-kata yang hilang, tapi juga adat dan identitas kita sebagai To Limola”*.

[Many children today no longer understand, so we must continue to use Limola so that they hear it and become accustomed to it. I also often invite young people to participate in traditional activities, such as harvest festivals or *motuno walundaka* (burning rice in bamboo), so that they can take part. Through this participation, they gradually understand the Limola language and will eventually pass it on. If this language disappears, it's not just the words that will be lost, but also our customs and identity as to Limola].

The elementary school principal emphasized the importance of young people's involvement in traditional activities. By encouraging them to participate in events such as harvest festivals and *motuno walundaka* (burning rice in bamboo), young people are expected to become accustomed to hearing and understanding the Limola language and eventually be able to use it. This strategy serves as an effective way to introduce the language in a real-life context, enabling young people to not only learn vocabulary but also absorb the cultural values embedded within it.

### The role of native speakers as language transmitters in the family environment

In addition to traditional leaders, native speakers (especially parents and the elderly) play an important role in revitalizing the Limola language. They are the primary agents in transferring the language to children through communication within the household. The language used in daily interactions, such as giving advice or even joking, is an effective means of naturally instilling Limola vocabulary and language structure. As revealed in an interview with an informant, AS (age 51):

“*kalau saya pribadi pak, sekarang tinggal Bersama istri, anak satu, kadang pakai bahasa Limola, kadang bahasa Indonesia, kadang bahasa Tae. Anak-anak belum terlalu tau, tapi pelan-pelan sudah menggali dan paham.”*

[Personally, sir, I live with my wife and one child. We sometimes use Limola, sometimes Indonesian, and sometimes the Tae language. My child is not very proficient yet, but is slowly beginning to understand and recognize it].

*Kalau anak Alhamdulillah sampai detik ini saya dominan pakai bahasa Limola, setiap saya komunikasi kebetulan ada anak saya di jawa, telfonan pakai bahasa Limola, chatingan pakai bahasa Limola*.

[Alhamdulillah, I still predominantly use Limola with my child to this day. Whenever we communicate, whether by phone or chat, I use Limola, even though my child lives in Java.]

Selanjutnya, hasil wawancara dengan SP mengungkapkan bahwa:

“*kadang saya pake bahasa Limola sama anakku yang kecil, kadang kalau ada saya suruhkan ambil sesuatu atau pergi beli sesuatu. Kalau anakku yang besar bisa semuami pakai bahasa limola, ini jhe yang kecil dia paham-pahammi sedikit”*.

[Sometimes I use Limola with my younger child, for example, when asking him to fetch or buy something. My older child can speak Limola fluently, while the younger one only understands a little.]

Based on interviews with AS and SP informants, native speakers, especially parents, play an important role in transmitting Limola to younger generations. Although Limola is not always used consistently in every situation, efforts to continue using the language in daily communication at home are an important strategy for revitalization.

### The role of the younger generation as successors through a digital approach

Findings show that the Sassa Village youth community has begun to actively participate in efforts to preserve the Limola language through social media. One of the platforms used is the Instagram account Pemuda Rumpun to Limola with the username @mosambuakeofficial. Through this media, they publish various activities related to preserving Limola culture and language. One of the most notable activities is the “*Dialog Kebudayaan to Limola”* (Cultural Dialogue to Limola), which raises the theme “*The Meaning of Traditional Food Symbols and the Limola Language.”* In addition, this platform also features short videos in Limola that involve the active participation of young people as a form of direct language inheritance. These activities signify the strong role of the younger generation in passing on language and culture to the next generation. This initiative reflects a growing awareness among young people that the preservation of regional languages can be done creatively and adaptively in line with technological developments, while also expanding the reach of the Limola language to a broader audience.

The social dynamics emerging from young people's involvement in preserving the Limola language indicate a shift in cultural transmission patterns from the traditional realm to the digital space. Although still minimal, this participation reflects great potential in language revitalization through approaches that are more contextual and relevant to the lives of the current generation. Social media such as Instagram has become a strategic tool for building collective awareness, strengthening cultural identity, and creating new spaces for interaction that involve young people as agents of change. This digital approach also allows language preservation to be carried out more inclusively, not limited to specific geographical areas, and opens up opportunities for collaboration across communities. This phenomenon marks a new social dynamic, where language preservation is not solely the responsibility of traditional leaders or formal institutions, but also part of participatory, creative, and adaptive community initiatives that embrace technological advancements.

Furthermore, the field findings from interviews regarding the role of youth in Sassa Village were also shared by one informant (RZ), currently a student. He stated that:

*Bahasa limola penting, karena sekarang bahasa Limola jarang yang pakai terutama pemuda, dia yang akan melanjutkan agar tidak punah. Namun tantangannya anu karena kurangnya tempat belajar. Saya tertarik Pak untuk melestarikan bahasa Limola, saya suka pakai bahasa Limola. Sekarang belum terlalu bisa, tapi saya bisa mengerti kalau ada berbahasa Limola. Kadang juga saya WA sama teman campur pakai bahasa Limola dan bahasa Indonesia*.

[The Limola language is important because it is rarely used nowadays, especially by young people, even though they are the ones who will carry on the language so that it does not become extinct. However, the challenge is that there are still very few places to learn it. I am personally interested in preserving the Limola language and enjoy using it. Although I am not yet very fluent, I can understand when others speak in Limola. Sometimes, I also communicate via WhatsApp with friends, mixing Limola and Indonesian.]

Young people play a key role in preserving the Limola language. As the group that will determine the future of this language, young people face significant challenges amid the tide of globalization and the dominance of national and foreign languages. Statements from informant RZ confirm that awareness of the importance of the Limola language has begun to grow among young people. They realize that if efforts are not made now, the Limola language is at risk of extinction due to the lack of active speakers, especially among the younger generation.

One of the main obstacles young people face is the lack of formal spaces or places to learn the Limola language. However, this challenge has encouraged various creative initiatives that utilize digital technology as a medium for learning and preservation. The digital approach is highly relevant because it can reach the younger generation, who are familiar with social media and technology.

A concrete example of this involvement can be seen in the Pemuda Rumpun to Limola community, which utilizes the Instagram platform (@mosambuakeofficial) as a space to publish cultural and language content. Activities such as the Limola Cultural Dialogue and short videos in Limola serve as a means to introduce vocabulary, sentence structure, and cultural values inherent in the language. Through these activities, the Limola language is not only learned as a means of communication but also as an identity closely tied to the traditions and values of the community.

Daily practices such as those shared by RZ, who uses the Limola language in online conversations, whether through chat or phone calls, are tangible contributions to preserving the language. Although its use is still limited to a mix with Indonesian, this effort demonstrates a genuine intention and commitment to continue introducing the Limola language in various modern communication contexts. Overall, these three themes reveal that the revitalization of Limola is a multi-scalar and multi-actor process. Traditional leaders safeguard symbolic and ritual domains, families sustain everyday transmission, and youth recontextualize the language within digital spaces. Together, these dynamics illustrate how community-based revitalization is negotiated across generational, spatial, and technological boundaries, offering a nuanced model for sustaining endangered languages in Indonesia and beyond.

## Discussion

Limola is one of the regional languages rich in cultural values and serves as the ethnic identity of the community in Sassa Village, Luwu Utara Regency, South Sulawesi. However, like many other local languages in Indonesia, this language is now facing the threat of extinction due to the declining number of active speakers, particularly among the younger generation. From a sociolinguistic perspective, this situation illustrates the dynamics described by Eliasson (2015) in his theory of Reversing Language Shift (RLS), which emphasizes that language endangerment often begins with the weakening of intergenerational transmission. The Limola case reflects Stage 7 of Eliasson scale, where the language still exists within ceremonial and family domains but is losing ground among youth in public and educational spheres. Addressing this requires targeted interventions that strengthen usage in both domestic and institutional contexts, preventing further progression toward language death. Mukhamdanah et al. (2025) state that a language is considered endangered when it is no longer transmitted to subsequent generations. To address this issue, the local community has initiated various revitalization efforts, focusing on preserving the functions of the Limola language within its traditional, social, and cultural contexts. This finding aligns with Budiono and Jaya (2024) research, indicating that the Limola language plays a significant role in the community's social structure, particularly in activities rooted in local values.

Through active community participation in cultural rituals, domestic interactions, and digital media, the Limola language indirectly remains an integral part of the daily lives of the To Limola community. This preservation strategy opens up an authentic space for the organic transmission of language from generation to generation. Such revitalization demonstrates that language preservation takes place through formal education and the strengthening of the social, spiritual, and symbolic functions of language within the community. These revitalization dynamics resonate with similar efforts observed in other language communities in Indonesia and globally. These dynamics also reflect Bourdieu (1991) concept of language as symbolic capital, where linguistic practices in ritual, domestic, and digital domains reproduce cultural authority, social cohesion, and group identity. The continued use of Limola in mopacci and motuno walundaka ceremonies, for example, reinforces the legitimacy of traditional leaders and embeds language within the moral fabric of community life. This shows that revitalization is not merely a linguistic task but a socio-political process that negotiates power, authenticity, and cultural continuity.

For instance, (Purnawati et al., 2025) highlights how the revitalization of the Balinese language was strengthened through its visibility in public spaces, such as the inclusion of Balinese script on signage and government buildings, which helped foster cultural pride and daily use. Likewise, Schafer et al. (2025) document how documenting Pagu proverbs in North Halmahera became a form of linguistic preservation deeply tied to cultural identity. These cases, much like Limola, demonstrate that revitalization is most effective when rooted in cultural practices and supported by symbolic visibility in everyday life. However, unlike the Balinese or Pagu cases, Limola revitalization is still primarily community-driven and has yet to receive strong institutional or policy support, indicating an urgent need for structural reinforcement to complement grassroots initiatives. As Pine and Turin (2017) emphasized, effective revitalization requires a holistic approach encompassing cultural, community, and technological domains, rather than merely formally preserving linguistic forms.

The use of Limola language in everyday social contexts, particularly among family members, neighbors, and fellow speakers, indicates its continued existence, albeit limited. This language tends to be used when both parties share the same ethnolinguistic background, while Indonesian is used in interactions with outsiders. Informant SP (age 46) explained, “If you are both Limola people, you will use Limola when you meet, unless there are outsiders who do not understand Limola, then you will use Indonesian.” Some outsiders who married residents eventually became able to use Limola after intensive interaction. This phenomenon reflects the flexibility and dynamism in the contextual use of language. Caira et al. (2025) and Annamyradova (2025) explain that language mixing in multilingual situations can strengthen social engagement while providing cognitive benefits. Specific communicative contexts for speakers can influence it. Thus, the use of Limola is not static but is greatly influenced by the context of interaction and patterns of social adaptation.

Efforts to revitalize the Limola language are also inseparable from the contributions of various elements of society. Traditional leaders are strategically guardians of oral traditions and collective culture. They are not merely leaders of traditional ceremonies, but also repositories of cultural knowledge manifested in stories, prayers, and life lessons. In practice, using the Limola language in rituals such as *mopacci* and *motuno walundaka* is a means of communication and a space for internalizing the values and ethnic identity of the To Limola people. As stated by Mr. SD, if this language disappears, what is lost is not merely a collection of words but the philosophy of life and the community's collective identity. As Pramuniati et al. (2025) mentioned, the loss of an ethnic language means the loss of the social-cultural identity of its speakers and the loss of cultural values accumulated by their ancestors. The resistance of traditional leaders to cultural homogenization is reflected in their decision to continue using Limola as the official language in traditional forums. Additionally, their involvement in encouraging the younger generation to participate in cultural activities is an important part of a language preservation strategy based on direct experience, which combines cognitive and affective aspects simultaneously.

In the domestic sphere, native speakers are important as language transmitters within the family. Informant AS, for example, continues to use Limola in long-distance communication with his child who lives in Java, even though the surrounding environment is more dominant in Indonesian and Tae. This practice demonstrates individual awareness and commitment to maintaining language continuity despite being in a multilingual environment. Similarly, informant SP uses Limola in functional situations with his children at home. His first child can already use the language actively, while his second child is just beginning to understand it. This indicates that the intensity and consistency of use in everyday contexts greatly influence the success of language transmission to younger generations. This finding supports the idea of Meighan (2024) that the primary goal of revitalization is to revive the language transmission process within families and communities, as the vitality of a language is largely determined by its usage in domestic settings (Tsunoda, 2006).

Meanwhile, the younger generation plays an important role as the language's successors through more adaptive and creative approaches. Although there is a tendency for Limola language usage to decline among teenagers, progressive initiatives have emerged from some young people to utilize digital media as a means of revitalization. Communities like *Rumpun To Limola* use Instagram to disseminate Limola-language content, ranging from short videos and cultural quotes to interactive dialogues. This approach aligns with Galla (2016) findings that digital media has strategic potential for documenting and promoting local languages. In the context of globalization, online forums can even become new spaces for minority language speakers to celebrate their identities openly and creatively (Ridanpää, 2018). However, digital media should not neglect the language's cultural values and spiritual context. Therefore, the younger generation needs to integrate elements of local wisdom into every content production to avoid simplification or distortion of the language's meaning. While digital initiatives expand the visibility and reach of Limola, they also raise important questions about authenticity and commodification. As Cahaya et al. (2022) notes, minority languages entering market-oriented digital spaces risk being reduced to consumable symbols detached from their deeper cultural significance. The challenge for Limola revitalization, therefore, is to ensure that technological adaptation remains anchored in cultural frameworks, balancing innovation with fidelity to traditional meanings.

Through an inclusive and participatory digital approach, young people are no longer merely recipients of cultural heritage but active agents of transformation, designing new ways to keep the language alive. They bridge traditional values with modern lifestyles, creating new spaces for the broader and more relevant use of the Limola language. With creativity and access to technology, they present a new face of language preservation that is more dynamic and reaches communities across borders. This signifies that efforts to revitalize the Limola language are no longer exclusive or top-down, but have become a collective movement born out of the collective awareness of the community, led by those who have a vision of cultural sustainability amid changing times. Thus, the sustainability of the Limola language is highly likely to be maintained if all elements of society continue to actively participate in language transmission, whether through traditional rituals, family communication, or culturally valuable digital innovations. Similar youth-led digital revitalization strategies have been observed internationally. The Māori language movement in New Zealand, for example, successfully harnessed social media campaigns and digital storytelling to normalize Māori in online spaces, complementing traditional “language nest” (kohanga reo) approaches that emphasize home and community use (Ruckstuhl, 2018). Likewise, Welsh revitalization efforts have shown how policy support for bilingual media content significantly expands language domains beyond ceremonial or domestic settings. Compared to these contexts, the Limola revitalization movement remains in its early stages, relying heavily on community initiatives such as the Rumpun To Limola Instagram platform. This difference highlights both the potential and limitations of grassroots-led efforts without systemic educational or policy frameworks. Integrating lessons from Māori and Welsh revitalization—particularly in terms of institutional backing and curriculum integration—could substantially enhance the sustainability and reach of Limola revitalization in the future.

From a policy perspective, the findings highlight the urgent need for multi-level interventions. At the local level, government and cultural agencies should support the inclusion of Limola in early childhood and primary education curricula, train teachers in culturally responsive pedagogy, and fund community-driven language documentation projects. At the regional level, policies could incentivize the production of bilingual media content and provide grants for digital storytelling initiatives led by youth groups. At the national level, recognition of Limola as part of Indonesia's intangible cultural heritage could attract institutional funding and ensure its integration into broader language planning frameworks. These measures would complement grassroots initiatives, bridging the gap between community efforts and structural support. Thus, this study advances the theoretical discourse on language revitalization by demonstrating how symbolic capital, intergenerational transmission, and digital mediation intersect in shaping the future of an endangered language. It also provides practical insights into how community-led revitalization can evolve into sustainable language policy when supported by institutional mechanisms.

## Limitations of the study

This study has several limitations that should be considered when interpreting the findings. First, the research was conducted in a single community—Sassa Village, North Luwu—which limits the generalizability of the results to other Limola-speaking areas or different sociolinguistic contexts in Indonesia. The dynamics of language revitalization may differ in communities with more institutional support, different demographic profiles, or varying levels of urbanization. Second, the study involved a relatively small number of participants (24 individuals) selected through purposive sampling, which, while appropriate for ethnographic depth, may not capture the full diversity of perspectives within the broader Limola-speaking population. Third, the Instagram content analysis was limited to publicly available posts from the Rumpun To Limola community page, which may not reflect the full spectrum of digital revitalization activities. Lastly, the study did not conduct longitudinal tracking of language use over time, so conclusions about long-term trends remain tentative. Future research should aim to expand the geographical scope, include larger and more diverse participant groups, and incorporate longitudinal methods to capture changes in revitalization practices and language vitality over time.

## Recommendations for policy and practice

The findings of this study point to several key recommendations for policymakers, educators, and community stakeholders:

1. Integrate regional languages into formal education—Local governments and education authorities should incorporate Limola and other regional languages into early childhood and primary education curricula, supported by culturally responsive teaching materials and teacher training programs.
2. Support community-driven documentation and revitalization—Funding and technical assistance should be provided for community-based projects documenting vocabulary, oral traditions, and cultural narratives, which are essential for sustaining linguistic diversity.
3. Leverage digital platforms strategically—Partnerships between cultural institutions and youth organizations can help develop culturally grounded digital content, expanding the presence of Limola in online spaces while preserving its symbolic meaning.
4. Recognize and protect traditional practices—Official recognition of ceremonies such as mopacci and motuno walundaka as elements of intangible cultural heritage would strengthen their role in intergenerational language transmission.
5. Promote intergenerational language spaces—Community centers, cultural festivals, and local events should intentionally create opportunities for older speakers and youth to interact using Limola, reinforcing language use across age groups.

Implementing these recommendations would not only support the sustainability of Limola but could also serve as a model for revitalizing other endangered languages in Indonesia, bridging grassroots initiatives with institutional support.

## Conclusion

This study shows that the Limola language plays a strategic role as a symbol of cultural identity and a medium of spiritual and social expression for the people of Sassa Village. Despite facing the threat of extinction due to language shift among the younger generation, revitalization efforts have been carried out in diverse and participatory ways. Traditional leaders continue to use the Limola Language in traditional rituals as a form of resistance against cultural homogenization. Native speakers, especially the elderly, play a crucial role in language transmission through daily communication in domestic settings. Younger generations are beginning to utilize digital technology as a creative tool to promote and preserve the Limola language in public spaces. These findings emphasize the need for integrated and community-based revitalization strategies that integrate traditional rituals, family environments, and modern technological adaptations. Thus, the revitalization of the Limola language not only ensures the language's survival and strengthens the cultural identity and resilience of the Sassa community in the face of globalization.

However, this study has several limitations that should be acknowledged. The research was conducted in a single village community, which may limit the generalizability of findings to other Limola-speaking areas or different endangered language contexts. The number of participants, although sufficient for thematic saturation, remains relatively small and may not capture the full diversity of perspectives in the broader community. In addition, the study relied heavily on self-reported narratives and participant observations within a limited timeframe, which may not fully reflect seasonal or long-term changes in language use patterns.

Future research could expand the geographical scope to include multiple Limola-speaking communities, enabling comparative analysis of revitalization strategies across different sociocultural settings. Longitudinal studies would also be valuable in tracking changes in language vitality and intergenerational transmission over time. Furthermore, integrating quantitative approaches, such as language proficiency surveys and social network analysis, could complement ethnographic insights and provide a more comprehensive understanding of the dynamics of language maintenance. Collaborative action research involving community members, educators, and policymakers is also recommended to design, implement, and evaluate intervention models that are culturally grounded and contextually sustainable.

## Data Availability

The original contributions presented in the study are included in the article/supplementary material, further inquiries can be directed to the corresponding author.

## References

[B1] Albury N. J. (2015). Objectives at the crossroads: critical theory and self-determination in indigenous language revitalization. Crit. Inq. Lang. Stud. 12, 256–282. doi: 10.1080/15427587.2015.1096732

[B2] Annamyradova A. (2025). A contrastive study of pragmatic and semantic features in typical and atypical comparative constructions across English, Chinese, Russian, and Turkmen: cognitive interpretations explored. Front. Educ. 10:1513434. doi: 10.3389/feduc.2025.1513434

[B3] Benu N. N. Artawa I. K. Satyawati M. S. Purnawati K. W. (2022). Local language vitality in Kupang city, Indonesia: a linguistic landscape approach. Cogent Arts Hum. 10:2153973. doi: 10.1080/23311983.2022.2153973

[B4] Bourdieu P. (1991). Language and Symbolic Power. Cambridge, MA: Harvard University Press.

[B5] Braun V. Clarke V. (2006). Using thematic analysis in psychology. Qual. Res. Psychol. 3, 77–101. doi: 10.1191/1478088706qp063oa

[B6] Budiono S. Jaya T. (2024). Evaluation of local language learning in the Limola language revitalization. J. Appl. Stud. Lang. 8, 20–30. doi: 10.31940/jasl.v8i1.20-30

[B7] Cahaya A. Yusriadi Y. Gheisari A. (2022). Transformation of the Education Sector during the COVID-19 pandemic in Indonesia. Educ. Res. Int. 2022, 1–8. doi: 10.1155/2022/8561759

[B8] Caira T. Declerck M. Struys E. (2025). Language-mixing in content and language integrated learning: benefit or burden? An auditory recall perspective. Front. Educ. 9:1520791. doi: 10.3389/feduc.2024.1520791

[B9] Crystal D. (2000). Language Death. Cambridge: Cambridge University Press.

[B10] Darquennes J. (2007). “Language contact and language conflict at the crossroads of cultures and languages: a Belgian perspective,” in Multilingualism in European Bilingual Contexts: Language Use and Attitudes, eds. D. Lasagabaster and A. Huguet (Clevedon: Multilingual Matters), 65–89.

[B11] Davis J. L. (2015). Language affiliation and ethnolinguistic identity in Chickasaw language revitalization. Lang. Commun. 47, 100–111. doi: 10.1016/j.langcom.2015.04.005

[B12] DePalma R. Zapico-Barbeito M. H. Sobrino-Freire I. (2018). Future teachers as agents of language revitalisation: the case of Galician early childhood education. Lang. Cult. Curric. 31, 303–317. doi: 10.1080/07908318.2018.1504402

[B13] Eisenlohr P. (2004). Language revitalization and new technologies: cultures of electronic mediation and the refiguring of communities. Annu. Rev. Anthropol. 33, 21–45. doi: 10.1146/annurev.anthro.33.070203.143900

[B14] Eliasson S. (2015). The birth of language ecology: interdisciplinary influences in Einar Haugen “The ecology of language” Lang. Sci. 50, 78–92. doi: 10.1016/j.langsci.2015.03.007

[B15] Farfan J. A. F. Cru J. (2021). Reviewing experiences in language (re)vitalisation: recent undertakings in the media and the arts. J. Multiling. Multicult. Dev. 42, 941–954. doi: 10.1080/01434632.2020.1827644

[B16] Galla C. K. (2016). Indigenous language revitalization, promotion, and education: function of digital technology. Comput. Assist. Lang. Learn. 29, 1137–1151. doi: 10.1080/09588221.2016.1166137

[B17] Gorter D. (2006). Introduction: the study of the linguistic landscape as a new approach to multilingualism. Int. J. Multiling. 3, 1–6. doi: 10.2307/jj.27939665.3

[B18] Gorter D. (2012). Minority language researchers and their role in policy development. Lang. Cult. Curric. 25, 89–102. doi: 10.1080/07908318.2011.653060

[B19] Grenoble L. A. Whaley L. J. (2006). Saving Languages an Introduction to Language Revitalization. Cambridge: Cambridge University Press.

[B20] Hatzikidi K. Lennox C. Xanthaki A. (2021). Cultural and language rights of minorities and indigenous peoples. Int. J. Hum. Rights 25, 743–751. doi: 10.1080/13642987.2020.1859487

[B21] Hermes M. Bang M. Marin A. (2012). Designing indigenous language revitalization. Harv. Educ. Rev. 82, 381–402. doi: 10.17763/haer.82.3.q8117w861241871j

[B22] Hinton L. Huss L. Roche G. (2018). The Routledge Handbook of Language Revitalization. London: Routledge Taylor and Francis Group.

[B23] Hornberger N. H. King K. A. (1996). Language revitalisation in the Andes: can the schools reverse language shift? J. Multiling. Multicult. Dev. 17, 427–441. doi: 10.1080/01434639608666294

[B24] Joseph J. E. (2004). Language and Identity: National, Ethnic, Religious. New York: Palgrave Macmillan Limited.

[B25] Khalsiah K. Yusuf Y. Q. Sobarna C. Sari D. F. Indirayani I. Azkiya A. (2025). Figurative expressions in Acehnese pregnancy cultural taboos as a language of protection. Indones. J. Appl. Linguist. 15, 147–160. doi: 10.17509/ijal.v15i1.79412

[B26] Language Development and Guidance Agency (2024). Indonesian Language Vitality Report. Jakarta: Ministry of Education, Culture, Research, and Technology.

[B27] McCarty T. L. (2018). “Community-based language planning: perspectives from indigenous language revitalization,” in The Routledge Handbook of Language Revitalization (Routledge), 22–35.

[B28] Meighan P. J. (2024). Indigenous language revitalization using TEK-nology: how can traditional ecological knowledge (TEK) and technology support intergenerational language transmission? J. Multiling. Multicult. Dev. 45, 3059–3077. doi: 10.1080/01434632.2022.2084548

[B29] Michael J. Chandler M. Lalonde C. (1998). Cultural continuity as a hedge against suicide in Canada's First Nations. Transcult. Psychiatry 35, 191–219. doi: 10.1177/136346159803500202

[B30] Mukhamdanah M. Zaman S. Khairiah D. Nurhuda P. Firdaus W. Hardaniwati M. (2025). Language use and attitudes of young speakers of Skou, Tabla, and Biak in Jayapura. Indones. J. Appl. Linguist. 15, 209–223. doi: 10.17509/ijal.v15i1.75114

[B31] Mulyawan I. W. (2021). Maintaining and revitalising Balinese language in public space: a controversial language planning regulation. Indones. Malay World 49, 481–495. doi: 10.1080/13639811.2021.1910356

[B32] Nurman Y. Yusriadi Y. Hamim S. (2022). Development of pluralism education in Indonesia: a qualitative study. J. Ethnic Cult. Stud. 9, 106–120. doi: 10.29333/ejecs/1207

[B33] Pine A. Turin M. (2017). “Language revitalization,” in The Oxford Handbook of Endangered Languages, eds. K. L. Rehg and L. Campbell (Oxford: Oxford University Press), 627–645.

[B34] Pramuniati I. Mahriyuni M. Hardini T. I. Hidayati F. (2025). Language shift between generations: regional-speaking parents raise Indonesian-speaking children in North Sumatra. Indones. J. Appl. Linguist. 15, 102–115. doi: 10.17509/ijal.v15i1.66704

[B35] Purnawati K. W. Artawa K. Satyawati M. S. Kardana I. N. (2025). Unveiling communication strategies through public space signs: a linguistic landscape study in Badung Smart Heritage Market, Bali-Indonesia. Cogent Arts Hum. 12:2444045. doi: 10.1080/23311983.2024.2444045

[B36] Redjeki D. S. S. Imron A. Rasdiana R. Wiyono B. B. Prestiadi D. Wahed A. . (2025). The nexus of Nusantara archipelagic cultural values in pupil management and its activities on social harmony through national identity revitalization. Front. Educ. 10:1524105. doi: 10.3389/feduc.2025.1524105

[B37] Ridanpää J. (2018). Why save a minority language? Meänkieli and rationales of language revitalization. Fennia 196, 187–203. doi: 10.11143/fennia.74047

[B38] Ruckstuhl K. (2018). Public policy and indigenous language rights: Aotearoa New Zealand's Maori Language Act 2016. Curr. Issues Lang. Plann. 19, 316–329. doi: 10.1080/14664208.2017.1391496

[B39] Schafer S. Syam M. Gogali L. (2025). Living together beyond liberal democracy: examples of local decision-making and managing resource extractivism in Indonesia. Front. Polit. Sci. 7:1370828. doi: 10.3389/fpos.2025.1370828

[B40] Tsunoda T. (2006). Language Endangerment and Language Revitalization: An Introduction. Berlin: Mouton de Gruyter.

[B41] UNESCO (2021). UNESCO Launches the World Atlas of Languages to Celebrate and Protect Linguistic Diversity. Paris: United Nations Educational, Scientific and Cultural Organization (UNESCO).

[B42] UNESCO (2023). Multilingual Education, the Bet to Preserve Indigenous Languages and Justice. Paris: United Nations Educational, Scientific and Cultural Organization (UNESCO).

[B43] White C. M. (2002). Language, authenticity and identity: indigenous fijian students and language use in schools. Lang. Cult. Curric. 15, 16–29. doi: 10.1080/07908310208666630

[B44] Wuttiphan N. Geng J. Robillos R. J. (2025). Enhancing Chinese as a Foreign Language (CFL) learners' listening comprehension skills through translanguaging within a pedagogical cycle. Pertanika J. Soc. Sci. Hum. 33, 1217–1238. doi: 10.47836/pjssh.33.3.13

[B45] Yulianti A. I. Hidayah A. M. N. Wibowo A. H. Maharani A. V. Septiana D. Rosni D. M. (2024). The Limola language and its syntax: unveiling the functions, structure, and categories. Indones. J. EFL Linguist. 365–378. doi: 10.21462/ijefl.v9i2.827

[B46] Yusriadi Y. Makkulawu Panyiwi Kessi A. Awaluddin M. Sarabani L. (2022). E-learning-based education resilience in Indonesia. Educ. Res. Int. 2022. doi: 10.1155/2022/7774702

